# A Comprehensive Review of *Ex-Vivo* Machine Perfusion in Uterus Transplantation

**DOI:** 10.3389/ti.2025.15254

**Published:** 2025-12-08

**Authors:** Eleni M. Drivas, Siavash Khaki, Amanda H. Loftin, Narges Lamsehchi, Liza Johannesson, Byoung Chol Oh, Gerald Brandacher

**Affiliations:** 1 Department of Plastic and Reconstructive Surgery, Johns Hopkins University School of Medicine, Baltimore, MD, United States; 2 Department of Pathology, Johns Hopkins University School of Medicine, Baltimore, MD, United States; 3 Department of Surgery, Annette C. and Harold C. Simmons Transplant Institute, Baylor University Medical Center, Dallas, TX, United States; 4 Department of Visceral, Transplant and Thoracic Surgery, Daniel Swarovski Transplantation Research Laboratory, Innsbruck Medical University, Innsbruck, Austria

**Keywords:** uterus transplantation, machine perfusion, machine preservation, *ex vivo* perfusion, vascularized composite allotransplantation

## Abstract

Uterine transplantation has revolutionized previously incurable causes of infertility. While most transplants are performed with live donors, the use of deceased donors could potentially expand the donor pool and increase the number of transplants performed. One limitation of deceased donor use is warm and cold ischemia time, which may be potentially mitigated by the implementation of *ex-vivo* machine perfusion (EVMP). This comprehensive review synthesizes the existing literature on uterine EVMP, highlighting both experimental and translational developments up to February 2025. A total of 31 relevant studies were identified from 244 screened articles, most involving human aor large-animal uteri. The majority of studies employed normothermic machine perfusion (NMP) as a model for physiologic conditions, focusing on endocrine or functional analysis, inflammatory reactions, or technical aspects of perfusion. Only in the past 6 years have articles looked at EVMP as a preservation technique for transplantation, or employed hypothermic machine perfusion (HMP). While EVMP has only recently increased in popularity for transplant preservation, uterine EVMP has historically been used in multiple studies as a model for physiologic conditions. While further research is needed to optimize preservation protocols, much can be gleaned from prior models of uterine perfusion.

## Introduction

Absolute uterine factor infertility is a significant cause of infertility, and was considered incurable until the last decade. Uterine transplantation represents a revolutionary approach to addressing infertility in women with absent or nonfunctional uteri, which may result from congenital uterine agenesis or hysterectomy due to malignant disease, postpartum hemorrhage, uterine fibroids, and congenital abnormalities [1]. Distinct from other solid organ transplants, uterine transplant poses unique challenges; it must not only be technically and immunologically feasible but also enable the transplanted uterus to sustain pregnancy and facilitate a healthy live birth [2].

The first human uterine transplant attempt was conducted in 2000 [3], but the first live birth occurred in 2014 in Sweden [4]. Since then, over 80 uterine transplants have been performed globally, resulting in more than 40 live births [5, 6]. Approximately 72% of the registered uterine transplants were from live donors, according to the United States Uterus Transplant Consortium (USUTC) and the International Society of Uterus Transplantation (ISUTx) [5, 6]. However, recent research has now focused on using deceased (brain-dead) donors as graft sources. While this would potentially increase the donor pool, it also requires more attention to organ preservation.

Despite advancements in uterus transplantation, significant knowledge gaps persist, necessitating further research in surgical techniques, immune modulation, and graft rejection studies [7]. Moreover, the effects of warm and cold ischemia in uterus transplantation are still not well understood. Uterine grafts have been shown to tolerate static cold ischemic storage (SCS) for at least 6 h while maintaining histologic integrity, ATP concentrations, and contractile ability [8].

One concern in the field of uterine transplantation is the tolerance of the uterus to ischemia, and the effects of warm and cold ischemia on graft viability and functionality. According to previous studies using animal models, the uterus exhibits a relative tolerance to both cold and warm ischemia [9, 10]. In the mouse model of uterus transplantation, it has been demonstrated that live births can be achieved following a cold ischemia duration of 24 h [11]. However, the optimal duration of cold ischemia for uterine grafts remains undetermined, necessitating further investigation. During the 24 h of cold storage of human uterine, Gauthier et al, demonstrated that no significant histomorphology changes had occurred in the tissue, and there was little evidence of apoptosis [12]. In the clinical setting, live births have resulted from both living and deceased donors, although living donors comprise the majority of live births [5, 6]. Deceased donors have a significantly longer cold ischemia time (CIT) as compared to living donors [5], but successful live births have resulted from CIT as long as 6.5 h [13] and 9 h [14]. Among uterus transplants in the United States, early graft loss was associated with longer warm ischemia time (WIT), but no association was found between CIT and clinical outcomes [5].

SCS has long been considered the gold standard for organ preservation [15]. However, advancements in *ex-vivo* machine perfusion (EVMP) technology, originally developed for solid organs, have opened new avenues for preserving a wider range of organs and delivering therapeutic agents [15]. Previous large-scale studies have indicated that EVMP may offer advantages over SCS in liver and kidney transplants, including improved patient survival rates, reduced adverse events, and enhanced short- and long-term functional outcomes [16, 17]. In addition, EVMP may offer several advantages, such as reducing cold ischemia and hypoxic injuries by ensuring a continuous supply of oxygen and nutrients, clearing toxic metabolites, and improving the quality and viability of the graft [18, 19].

In clinical settings, this technique is now frequently applied for lung, heart, liver, and kidney transplantation [20–22]. In particular, EVMP has shown potential for extremity vascularized composite allotransplantation (VCA), in which static cold storage typically requires reperfusion within 10–12 h to maintain viability, with optimal functional recovery anticipated between 3 and 6 h of cold ischemia [23]. The implementation of EVMP may be particularly important in uterus transplantation due to the non-vital nature of the uterus, which often leads to prolonged CIT during multi-organ procurement surgeries, as hysterectomies are performed as the last procedure in some protocols [24, 25].

Despite the growing fields of research in both uterus transplantation and EVMP, no comprehensive review papers exist on uterus machine perfusion. The purpose of this study is to conduct a extensive review of literature on uterus *ex-vivo* machine perfusion, including identification of relevant literature, characterization of these studies in terms of perfusion protocol and outcomes, and comparison of protocols.

### Search Approach and Evidence Selection

A comprehensive literature search of manuscripts listed in PubMed, Scopus, EMBASE, Cochrane Library, and ClinicalTrials.gov databases was conducted in January 2025. The following search terms were used: [(uterus) OR (uteri) OR (uterine)] AND [(machine perfusion) OR (machine preservation) OR (*ex vivo* perfusion) OR (extracorporeal perfusion) OR (extracorporeal circulation)]. Selected studies met the following inclusion criteria: (1) preclinical articles studying machine perfusion; (2) perfusion of uterus grafts; (3) randomized control trials, prospective and retrospective case-control and cohort studies, cross-sectional cohort studies, case reports, and technique papers. Exclusion criteria were: (1) reviews without presentation of new data; (2) abstracts, conference papers, editorials, or comments; (3) articles about solid-organ or non-uterine VCA perfusion. Historically, however, perfusion systems have been extensively used for physiological and hormonal studies of the uterus, providing a valuable foundation for the future development of this approach in organ preservation. Despite the heterogeneity among existing studies, we included all such research in our review to capture the full scope of relevant evidence.

**FIGURE 1:**
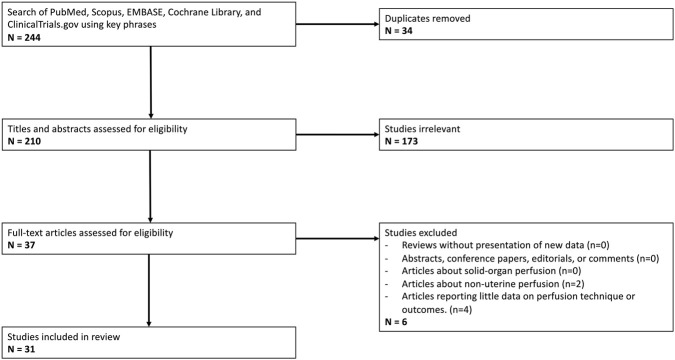
PRISMA Flow Diagram outlining inclusion and exclusion criteria, number of abstracts screened, and full texts retrieved.

The literature search yielded 244 articles, of which 31 articles met criteria (see Figure 1; Table 1) [7, 26–33, 35–56]. Included studies were published between 1970 and 2025. Ten studies utilized human uteri, while the remaining 21 used animal models. Of these animal models, all but one were in large animals, with swine being the most common (15 studies). Other animals included sheep (2 studies), cows (2 studies), and horses (1 study). Only one study [28] used a small animal model (rabbits), and no studies used rodents.

**TABLE 1:** Summary of reviewed papers, n = 31.

Author (Year)	Species (Details)	Surgical Details	Cannulation Details	Temp (°C)	Duration (Hr)	Flow (mL/min)	Pressure (mmHg)	Perfusion Pump Setup	Perfusate Details	Perfusion Monitoring	Study Design (# of uteri)	Outcomes
Peirce [26]	Sheep (38–62 kg, near-term)	Pregnant uterus removed and placed in “artificial abdomen”	Bilateral uterine arteries	37	0.5–5	300+	NR	2 roller pumps, artificial membrane lung for oxygenation	Heparinized maternal blood	Perfusate chemistry and gas	NMP [15]	Early fetal death early in all but 5 perfusions, survival up to 5 h in one experiment
Tojo [27]	Human	Hysterectomy for benign disease and trophoblastic tumor	Bilateral uterine arteries	37	5	25–40	NR	1 diaphragm pump with Y-connector, oxygenator with oxygen	Hank’s solution, 20% autologous whole blood, 4% dextran	Perfusate chemistry and gas, EMG uterine muscle, biopsy tumor tissue, angiogram after perfusion	NMP [2]	Viability up to 5 h, preservation of trophoblastic tumor *in utero*
Bloch [28]	Rabbit (3–4 kg)	Single uterine horn included, contralateral blood supply ligated	Aorta	37	7–10	6–8	NR	1 pump, oxygenator with carbogen	Krebs-henseliet buffer, dextran	Perfusate prostaglandin levels	NMP with angiotensin II, oxytocin, epinephrine, and arachidonic acid [29]	Increased prostacyclin release with all substances, most effectively for arachidonic acid
Bulletti [30]	Human	Scheduled hysterectomies for benign and malignant diseases	Bilateral uterine arteries and veins, 16G	37	12	10–30	80–120	2 roller pumps, oxygenator with carbogen	KRBB, heparin	Perfusate chemistry and gas, uterine biopsy	NMP [9]	Viability up to 12 h
Bulletti [31]	Human	Scheduled hysterectomies for benign and malignant diseases	Bilateral uterine arteries and veins, 16G	37	48	12–35	80–120	2 roller pumps, oxygenator with carbogen	KRBB, heparin	Perfusate chemistry and gas, uterine biopsy	NMP [20]	Viability up to 48 h, tissue is responsive to estrogen and progesterone
Bulletti [32]	Human	Scheduled hysterectomies for benign and malignant diseases	Bilateral uterine arteries and veins, 16G	37	52	18–30	80–120	2 roller pumps, oxygenator with carbogen	KRBB, heparin	Uterine biopsy	NMP after injection of fertilized embryo [3]	Successful implantation and trophoblastic invasion after 52 h in one of three uteri
Bulletti [33]	Human	Scheduled hysterectomies for benign and malignant diseases	Bilateral uterine arteries and veins, 16G	37	1	30	120	2 roller pumps, oxygenator with carbogen	KRBB, heparin	Perfusate estrogen levels, uterine biopsy	NMP with radio-labeled compounds to assess estrogen uptake [34]	Differential permeability of uterine vascular beds during proliferative and secretive phases
Bulletti [35]	Human (36–42 years)	Scheduled hysterectomies for benign and malignant diseases	Bilateral uterine arteries and veins, 16G	37	1.5, 48	NR	NR	2 roller pumps, oxygenator with carbogen	KRBB, heparin	Perfusate chemistry and gas, IUP, EMG uterine muscle	NMP for 48 h [3] NMP for 1.5 h with estrogen [5], estrogen/progesterone [5]	No spontaneous muscle activity in control uteri, increased muscle activity with estrogen, decreased with progesterone
Richter [36]	Human (28–56 years)	Scheduled hysterectomies for benign diseases	Bilateral uterine arteries, 14G	37	24	15–35	70–130	2 roller pumps, oxygenator with carbogen	Modified KRBB^a^, gentamicin	Perfusate chemistry and gas, uterine biopsy	NMP without exchange [5] NMP with exchange every 1 h [5], 2 h [5], 4 h [5], 6 h [5]	Increased damage with exchange every 6 h, viabillity in 1–4h groups
Baumer [37]	Cow (2+ years)	Post-mortem excision, 30–45 min WIT	Bilateral uterine arteries and veins	39	5	12–17	NR	1 peristaltic pump with Y-connector, oxygenator with carbogen	Autologous whole blood plus tyrode solution (4:1 ratio), heparin	Perfusate chemistry and gas, uterine biopsy	NMP [4] NMP with addition of Lugol’s solution [4], arachidonic acid [5]	Viability up to 5 h, adequate inflammatory response to irritants
Dittrich [38]	Swine (5–18 months)	Post-mortem excision	Bilateral uterine arteries and veins, 16–24G	37	7	15	100	2 roller pumps, oxygenator with carbogen, no recirculation	Modified KRBB^a^, calcium carbonate	Perfusate chemistry and gas, IUP	NMP with oxytocin [15], PGE2 [15]	Viability up to 7 h, contractions induced by both oxytocin and PGE2
Richter [29]	Human (34–46 years)	Scheduled hysterectomies for benign diseases	Bilateral uterine arteries, 14G	37	27	15–35	70–130	2 roller pumps, oxygenator with carbogen	Modified KRBB^a^	Perfusate chemistry and gas, uterine biopsy	NMP with oxytocin [5], estradiol/oxytocin [5]	Increased oxytocin receptor concentration in estradiol/oxytocin group compared to oxytocin alone
Richter [39]	Human (31–46 years)	Scheduled hysterectomies for benign diseases	Bilateral uterine arteries, 14G	37	27	15–35	70–130	2 roller pumps, oxygenator with carbogen	Modified KRBB^a^	Perfusate chemistry and gas, uterine biopsy	NMP [5] NMP with oxytocin [5], estradiol/oxytocin [5]	Increased oxytocin receptor gene expression in estradiol/oxytocin group compared to oxytocin alone
Braun [40]	Cow (2+ years)	Post-mortem excision, 75 min WIT	Bilateral uterine arteries and veins	39	6	17	NR	1 peristaltic pump with Y-connector, oxygenator with carbogen	Autologous whole blood plus tyrode solution (4:1 ratio), heparin	Perfusate chemistry and gas, uterine biopsy	NMP [6] NMP with addition of arachidonic acid [18]	Viability up to 6 h, increased inflammatory markers in arachidonic acid exposure group
Maltaris [41]	Swine (5–18 months)	Post-mortem excision	Bilateral uterine arteries, 16–24G	37	8	15	100	2 roller pumps, oxygenator with carbogen	Modified KRBB^a^	Perfusate chemistry and gas, IUP	NMP with acetylsalicylic acid [5], atosiban [5], ethanol [5], fenoterol [5], ritodrine [5], terbutaline [5], propofol [5], glyceryl trinitate [5], verapamil [5]	Increased contractility with all substances, most effectively with fenoterol
Mueller [42]	Swine (5–18 months)	Post-mortem excision	Bilateral uterine arteries, 16–24G	37	8	15	100	2 roller pumps, oxygenator with carbogen	Modified KRBB^a^, oxytocin added to induce contractions	Perfusate chemistry and gas, IUP	NMP with estrogen [34], progesterone [34], estrogen/progesterone [34]	Estrogen increased contracility, progesterone antagonized effects of estrogen
Mueller [43]	Swine (5–18 months)	Post-mortem excision	Bilateral uterine arteries, 16–24G	37	8	15	100	2 roller pumps, oxygenator with carbogen	Modified KRBB^a^	Perfusate chemistry and gas, IUP in corpus and isthmus	NMP with PGF2a [15], PGE1 [15], PGE2 [15], oxytocin [15]	Increased IUP globally with oxytocin and PGF2a, IUP gradient (isthmus > corpus) with PGE1 and PGE2
Mueller [44]	Swine (5–18 months)	Post-mortem excision	Bilateral uterine arteries	37	8	15	100	2 roller pumps, oxygenator with carbogen	Modified KRBB^a^, oxytocin added to induce contractions	Perfusate chemistry and gas, bilateral IUP	NMP with unilateral addition of estrogen [20], progesterone [20], estrogen/progesterone [20]	Estrogen increased contractility in ispilateral horn but not contralateral, progesterone antagonized effects of estrogen
Kunzel [45]	Swine (5–18 months)	Post-mortem excision	Bilateral uterine arteries, 16G	37	8	15	100	2 roller pumps, oxygenator with carbogen	Modified KRBB^a^, oxytocin added to induce contractions	Perfusate chemistry and gas, IUP	NMP with butylscopolamine [12], atropine [13], denaverine [15], morphine [7], metamizole [9], pethidine [10], celandine [14]	Decreased contractility with all substances, most effectively for denaverine
Dittrich [46]	Swine (5–18 months)	Post-mortem excision, division into two horns for simultaneous perfusion	Bilateral uterine arteries, 16–24G	37	3.5	NR	NR	2 roller pumps, oxygenator with carbogen	Modified KRBB^a^, oxytocin added to induce contractions	Bilateral IUP	Simultaneous NMP of bilateral horns with unilateral addition of human seminal plasma [17]	Improved contractility on human seminal plasma side
Geisler [47]	Swine (5–18 months)	Post-mortem excision	Bilateral uterine arteries, 16G	37	24	15	NR	2 roller pumps, oxygenator with carbogen	KRBB or modified KRBB^a^, oxytocin added to induce contractions	Perfusate chemistry and gas, IUP	NMP with KRBB [11], modified KRBB^a^ [18], modified KRBB with exchange every 2 h [11]	Improved contractility with modified KRBB^a^, viability up to 17 h with perfusate exchange
Kunzel [48]	Swine (5–18 months)	Post-mortem excision	Bilateral uterine arteries, 16G	37	NR	15	80–100	2 roller pumps, oxygenator with carbogen	Modified KRBB^a^	Perfusate chemistry and gas, IUP	NMP with PGE1 [3], PGE2 [3], PGF2a [3], progesterone/PGE1 [18], progesterone/PGE2 [16], progesterone/PGF2a [15]	Prostaglandin-induced contractions reduced by progesterone
Stirland [49]	Human	Scheduled hysterectomies for fibroids	Bilateral uterine arteries	38	8	NR	100	1 peristaltic pump with Y-connector, oxygenator with carbogen	Krebs-henseleit buffer, heparin, gentamicin, insulin, glutathione	Perfusate chemistry and gas, uterine biopsy	NMP with methylene blue [14]	Poor methylene blue staining in fibroids
Oppelt [50]	Swine (5–18 months)	Post-mortem excision	Bilateral uterine arteries, 24G	37	4	10–15	60–80	2 roller pumps, oxygenator with carbogen	Modified KRBB^a^	Perfusate chemistry and gas, IUP in corpus and isthmus	NMP control [18] NMP with progesterone [26], dienogest [38]	Progesterone decreased contractility globally, dienogest decreased contractility at ithmus only
Weinschenk [51]	Swine (7–18 months)	Post-mortem excision, 20min WIT	Bilateral uterine arteries, 16G	37	1	6	NR	2 roller pumps, oxygenator with carbogen	Modified KRBB^a^	IUP	NMP with procaine [31], lidocaine [31], ropivacaine [32]	Lidocaine and ropivacaine reduce contracitlity in higher concentrations
Padma [7]	Sheep (9–12 months)	Post-mortem excision	Bilateral uterine arteries, 26G	37	48	NR	45–55	1 peristaltic pump with Y-connector, oxygenator with carbogen	DMEM/F-12, GlutaMAX, fetal bovine serum, antibiotic-antimicotic solution	Perfusate chemistry and gas, uterine biopsy	SCS 4 h then NMP 48 h [6] SCS 48 h then NMP 48 h [7]	Reperfusion damage in 48 h storage but not 4 h storage
Kohne [52]	Horse (8–25 years)	Post-mortem exicsion after exsanguination, 60–100 min WIT	Bilateral uterine and ovarian arteries, 14–18G	39	8	30	NR	1 peristaltic pump with 3 Y-connectors, oxygenator with oxygen	Autologous whole blood plus autologous plasma (3:2 ratio), heparin	Perfusate chemistry and gas, uterine biopsy, sonomicrometry	NMP [12]	Viability up to 6 h, decreased function after 4 h s
Dion [53]	Swine	Uterus removed *en bloc* with aorta and IVC	Aorta	4	18	NR	NR	VitaSmart machine perfusion system (1 peristaltic pump)	UW solution	Macroscopic assessment	HMP 18h then transplant (NR)	Viable transplant
Loiseau [54]	Swine (150 kg)	Post-mortem excision, 60min WIT, uterus removed *en bloc* with aorta and IVC	Aorta	4 (HMP) or 37 (NMP)	12 (HMP) or 2 (NMP)	NR	15 (HMP) or 30–35 (NMP)	VitaSmart machine perfusion system (1 peristaltic pump) (HMP) or liverassist machine perfusion system (1 peristaltic pump) (NMP)	UW solution (HMP) or heparinized autologous whole blood (NMP)	Perfusate chemistry and gas, uterine biopsy	SCS 12h then NMP 2h [5] HMP 12h then NMP 2h [5]	Decreased resistance indices and higher tissue oxygenation during reperfusion in HMP group as compared to SCS
Cabanel [55]	Swine (30–40 kg)	Post-mortem excision, less than 60min WIT	Bilateral uterine arteries, 18G	20	4	2.5–10	25–35	2 roller pumps, oxygenator with carbogen	Steen+ solution	Perfusate chemistry and gas, serial weights, post-perfusion angiography	SNMP [4]	Viability for 4 h perfusion, stable weight throughout perfusion, well-identified microvasculature post-perfusion
Sousa [56]	Swine	Uterus removed *en bloc* with aorta and IVC	Aorta	4	18	NR	3	VitaSmart machine perfusion system (1 peristaltic pump)	UW solution	Macroscopic assessment, uterine biopsy, post-transplant blood samples	SCS in HTK 18h then transplant [5] SCS in UW 18h then transplant [5] HMP 18h then transplant [5]	Improved histology after transplant in HMP group initially but equivocal after 3 h, no biomarkers for uterus viability identified

NR, not recorded; WIT, warm ischemia time; NMP, normothermic machine perfusion; SNMP, sub-normothermic machine perfusion; HMP, hypothermic machine perfusion; SCS, static cold storage; IUP, intrauterine pressure; KRBB, Krebs-Ringer bicarbonate buffer.

^a^ Modified KRBB, Krebs-Ringer bicarbonate buffer with added saccharose, glutathione, dithiothreitol, 50 IU/L regular insulin.

### Experimental Focus

The included studies comprise a variety of experimental aims. As machine perfusion has only recently increased in popularity for organ preservation, many of the total published works on uterus machine perfusion do not have an end goal of transplantation or organ preservation. The most common experimental aim was endocrine and/or functional analysis (16 studies), which involved contraction monitoring and biochemical analysis after the administration of various hormones, drugs, or prostaglandins. Another portion of studies focused on technical aspects of the perfusion (5 studies), including perfusate composition, perfusion flow and pressure, and the influence of perfusate exchange. Four studies analyzed EVMP as a storage method, comparing machine-perfused uteri to uteri stored statically on ice. Of these four studies, two included subsequent transplantation. Other experimental aims included EVMP as a model for inflammatory reactions (2 studies), preservation of a pregnant sheep uterus (1 study), preservation of an intrauterine trophoblastic tumor (1 study), *in vitro* fertilization of a machine-perfused uterus (1 study), and analysis of fibroid blood supply via addition of methylene blue (1 study).

### Surgical Technique and Anatomical Considerations

**FIGURE 2:**
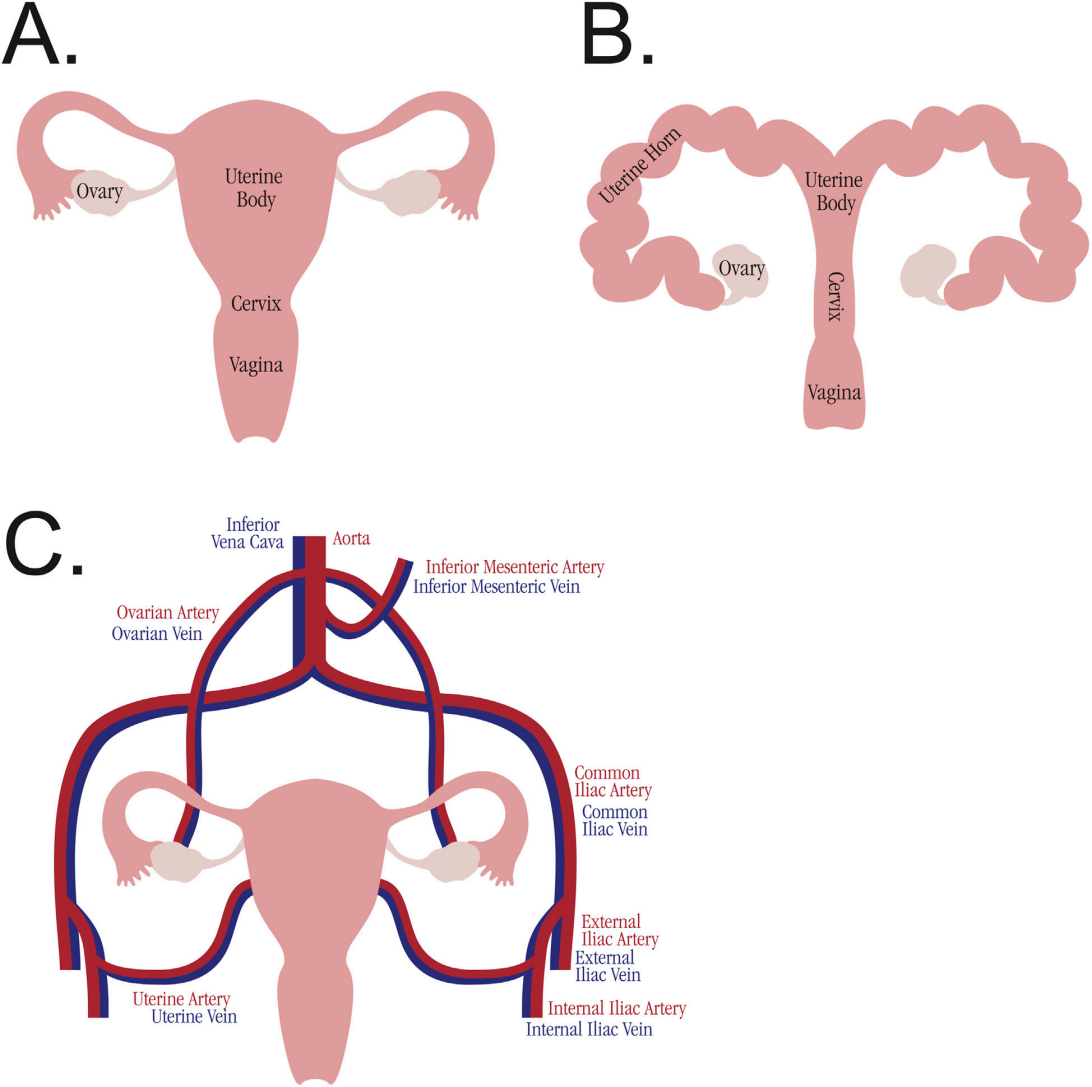
Comparative uterine anatomy. **(A)** Anatomy of the human uterus. **(B)** Anatomy of the swine uterus. **(C)** Blood supply to the uterus and ovaries, analogous across species.

The uterus is relatively unique in its blood supply as compared to other transplants (see Figure 2). The majority of other solid organ transplants and VCAs have a single-artery and single-vein blood supply, allowing for simplified machine perfusion with a single roller pump. The body of the uterus (as well as the uterine horns in large animal anatomy) is perfused via bilateral uterine arteries, which arise from the bilateral internal iliac arteries. They drain via bilateral uterine veins (also referred to in humans as inferior uterine veins) [57], which drain into the bilateral internal iliac veins. The ovaries have a separate blood supply, bilateral ovarian arteries and veins, which originate directly from the aorta and drain directly into the inferior vena cava, respectively. In humans, the distal ovarian vein is referred to as the superior uterine vein, and accounts for a large portion of uterine venous drainage [57]. The majority of studies (27 studies) cannulated the bilateral uterine arteries. Of these studies, eight also cannulated the uterine veins bilaterally. One study cannulated both the uterine and ovarian arteries bilaterally, although the ovarian arteries do not provide a significant blood supply to the uterine body and are not utilized for anastomosis in uterine transplantation [57]. While the majority of studies kept the uterine body intact, one study divided the uterus along the midline to perfuse both sides simultaneously [46]. Another study, the only one to use a small animal model [28], isolated a single uterine horn and cannulated it through the abdominal aorta, ligating all other arterial branches. Three recent studies [53, 54, 56] use a technique of removing the uterus *en bloc* with the abdominal aorta and inferior vena cava, thereby allowing for a single cannulation site at the aorta for perfusion of both uterine arteries and both ovarian arteries. A number of studies (17 studies) removed the uterus after euthanasia, most commonly in the setting of sourcing research animals from slaughterhouses.

### Perfusion Machine Design

**FIGURE 3:**
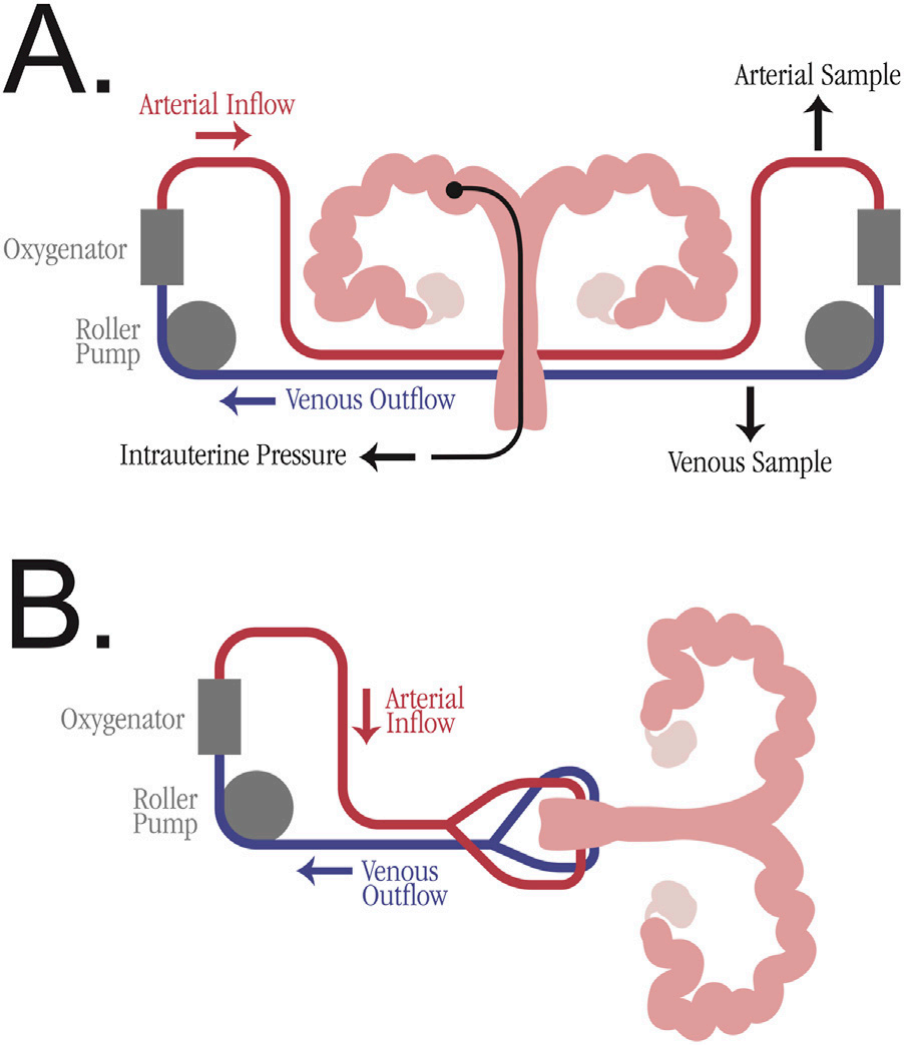
Simplified perfusion machine diagrams for uterus machine perfusion. **(A)** Individual arteriovenous circulation model with two roller pumps and oxygenator. Potential sampling venues are marked, including arterial perfusate sample, venous perfusate sample, and intrauterine pressure sample. **(B)** Mixed arteriovenous circulation model with one roller pump and a Y-connector for division of perfusate into bilateral uterine arteries.

The bilateral blood supply of the uterus poses a challenge for traditional machine perfusion devices, which typically have a single arterial inflow and single venous outflow. While some studies utilized a Y-connector after a single perfusion pump (6 studies), the majority of studies employed two separate pumps (21 studies), enabling adjustments to each artery individually and preventing unequal flow (see Figure 3). The studies cannulating the aorta all used a single pump for perfusion. All studies employed oxygenation of the perfusate, with the majority (28 studies) using carbogen gas. The perfusate medium was blood-based in 6 studies, but the majority of studies employed various organ preservation solutions, including Krebs-Ringer bicarbonate buffer, Krebs-Henseleit buffer, and UW solution. Multiple studies utilized perfusate additives, including heparin, antibiotics, and insulin. The duration of the perfusions varied, with 11 studies perfusing for 1–6 h, 11 studies perfusing for 6–12 h, and 10 studies perfusing for more than 12 h. The longest perfusion was 52 h, utilizing a non-blood-based perfusate [32].

As in solid organ machine perfusion, there is no consensus for the optimal temperature of uterus EVMP. As the majority of studies were not focused on storage or transplantation, most studies (28 studies) employed normothermic machine perfusion (NMP) (37 °C–39 °C) to mimic physiologic conditions. Three recent studies on uterine transplant preservation analyzed hypothermic machine perfusion (HMP) (4 °C) [53, 54, 56], and a fourth recent study on preservation analyzed sub-normothermic machine perfusion (SNMP) (20 °C) [55].

Flow and pressure varied greatly among studies that reported these values. These studies encompass perfusion of uteri from both humans and a variety of large animal models, thereby representing a wide range of uterine sizes. However, many studies (16 studies) employed normotensive or near-normotensive pressures (approximately 80–120 mmHg). Three recent studies [54–56], all in swine and all focused on transplant preservation, utilized much lower pressures (15–35 mmHg), aiming to mimic the low pressures employed in pancreas machine perfusion.

### Graft Monitoring

In addition to flow, pressure, and temperature monitoring, there are multiple methods for assessing the uterine graft during and after machine perfusion. The majority of studies (24 studies) collected perfusate samples for chemistry and gas analysis, looking at changes in pH, pCO_2_, pO_2_, bicarbonate, potassium, and lactate over time. Many studies calculated change in uterine weight to assess edema during perfusion. The structural integrity of the uterus was assessed through various methods, including uterine biopsy, macroscopic appearance of the graft, and post-perfusion angiography. Multiple studies also utilized methods to assess the functional status of the organ, most commonly with the measurement of intrauterine pressure, but also through electromyography of the uterine muscle. Intrauterine pressure catheters allowed for the measurement and calculation of uterine contractions, many of which were induced artificially by the addition of oxytocin or prostaglandins.

## Discussion

### Surgical Model

The uterus poses unique challenges in the implementation of successful EVMP, especially in preclinical animal models. While the swine uterus was the most common animal model utilized, the anatomy is not identical to humans (see Figure 2). Furthermore, animal size and age both influence the uterine graft size and therefore the caliber of the arteries cannulated. Studies utilizing larger animals such as cows, or animals which had previously given birth, reported cannulating the uterine arteries with 14G or 16G catheters. Other studies which used 6 to 18-month-old swine reported uterine artery cannulation with catheters as small as 24G. Swine do not typically start to sexually mature until at least 7–8 months of age [58], so the use of younger swine further limits the uterine size. The one study to utilize a small animal model cannulated the aorta of the rabbit, presumably due to the uterine arteries being too small to cannulate. Even if cannulated, small-caliber arteries may not be amenable to successful anastomosis during subsequent transplantation, especially with the multiple vessel anastomoses required for uterine transplant. Therefore, animal size and age should be taken into serious consideration when choosing a model species.

One method for circumventing the surgical challenge of small-caliber arteries and multiple transplant anastomoses is to remove the uterus *en bloc* and cannulate via the aorta, which is described in three recent studies [53, 54, 56]. This technique has been previously described in preclinical uterine transplant models [58, 59]. In addition to reducing the anastomosis and cannulation site to a single large-caliber artery, this technique also incorporates the bilateral ovarian arteries and veins, which are often excluded from uterine EVMP models. However, this technique is surgically challenging, requiring the skeletonization of the aorta and its bifurcation, the inferior vena cava (IVC) and bifurcation, and the bilateral iliac vessels. All non-utero-ovarian branches from the infrarenal aorta, infrarenal IVC, and bilateral internal iliac vessels must be identified and ligated. The rectum or sigmoid colon must be transected in order to remove the uterine blood supply *en bloc*. The studies utilizing this model flushed the uterus with cold preservation solution retrograde through an external iliac artery (while clamping the infrarenal aorta), prior to definitive dissection of the uterine vessels. The reason for this is twofold: to minimize warm ischemia time during a lengthy dissection, and to mimic human deceased donor uterine procurements, in which the uterus is removed after all other essential organs are procured. Overall, this method for uterine procurement can be beneficial, especially if working with a smaller or younger animal model, but it requires an experienced surgical team and complex anatomical knowledge. This method is also limited to being performed as a terminal procedure and allotransplant model, preventing the utilization of an autotransplant model.

### Optimal Perfusion Protocol

Given the breadth of variables involved in EVMP, it is difficult to devise an optimal perfusion protocol. Among VCA EVMP, there is no consensus on temperature or perfusate composition, although multiple studies have shown its benefit when compared to SCS [34, 60]. However, synthesis of the reviewed studies can identify some best practices for implementing EVMP in a uterine graft. The use of two perfusion pumps with individual pressure and flow adjustments is preferable to a single pump with a Y-connector (see Figure 3). This dual-pump system prevents unequal flow in the bilateral arteries due to variable pressure gradients [55]. In addition to oxygenation with carbogen, a perfusion medium should be prepared containing an organ preservation solution. While some studies added autologous whole blood to the perfusion medium, this may be impractical in clinical translation for a multi-organ deceased donor procurement.

The goal perfusion pressure varied between studies. The majority of large animal non-uterine VCA perfusions utilize normotensive pressures (60–80 mmHg) [34, 60], and many of the reviewed uterine studies reported similar goal pressures. However, three recent swine studies [54–56] utilized lower pressures (15–35 mmHg), citing the small caliber of the vessels and modeling the protocol after pancreas machine perfusion. Further research is needed to determine the optimal perfusion pressure, which likely will depend on animal size and vessel caliber.

As in solid organ EVMP, there is no consensus for optimal perfusion temperature in non-uterine VCA EVMP [15, 34, 60]. The articles reviewed in this paper predominantly utilize NMP, as many are using EVMP to model physiologic conditions rather than as a preservation method. Additional research into uterine HMP and SNMP is required to determine if lower temperatures are beneficial for uterine graft preservation.

### Advantages and Limitations of *Ex-Vivo* Machine Perfusion in Uterus Transplantation

EVMP offers several potential advantages over static cold storage in the context of uterus transplantation. First, it may provide prolonged preservation times beyond those available through cold storage [61]. EVMP is able to continuously monitor perfusion parameters, such as flow, pressure, and metabolic activity, which may provide valuable insight into the viability of grafts prior to transplantation [61, 62]. Furthermore, it provides a therapeutic platform that helps to attenuate ischemia-reperfusion injury by providing oxygenated perfusate and targeted pharmacological or immunomodulatory interventions during preservation [62]. By enabling real-time evaluation of perfusion dynamics, EVMP may support viability testing and help identify uterine grafts with the greatest likelihood of successful transplantation.

Although EVMP has demonstrated promising results for solid organ preservation, the supporting evidence for its use in uterus transplantation remains preliminary because most studies have been conducted in animal models with limited human experience; therefore, its benefits for uterine preservation have not yet been conclusively determined. Perfusion systems, on the other hand, are expensive, technically complex, and require specialized knowledge [63]. In addition, perfusion itself may introduce risks, such as mechanical injury to delicate vascular endothelium or oxidative stress due to inadequate oxygenation [64].

### Future Applications

As a whole, EVMP has the potential to not only prolong storage of uterine grafts, but also to optimize the graft itself. In solid organs, EVMP has been shown to recondition non-acceptable organs to be successfully transplanted [65, 66], thereby increasing organ availability and expanding the donor pool. The potential for extended storage times, organ optimization, and even immune engineering make EVMP a promising future technology for the practice of uterus transplantation.

### Limitations and Suggestions for Future Research

This review is presented with the acknowledgement of several limitations. The literature search was conducted under the assumption that all relevant articles would be identifiable by the designated search terms and the databases utilized. Additionally, the review excluded abstracts, conference presentations, and unpublished data. There is a possibility that significant and noteworthy research on uterine EVMP was not included in the literature review, which might have allowed further insight into this topic.

Many of the articles discussed in this paper were published over 10 years ago. These studies did not have access to the most up-to-date protocols or designs of EVMP, especially as this is a rapidly-evolving technology. Therefore, the methods discussed in these articles may be outdated and not applicable to modern uterine transplantation practices. Only four articles, all published within the past 6 years, looked at EVMP as a method for transplant preservation. This small sample size makes it difficult to generalize and translate the studies into clinical practice. More studies on EVMP as a preservation method, especially HMP and SNMP, are needed to further research on this topic.

Despite its potential benefits, questions remain regarding the definitive effects of EVMP on uterus transplantation. In contrast to all other organs, uterine transplants are temporary, with hysterectomies performed after the birth of one or two children. Therefore, the improved long-term graft function associated with EVMP may be of lesser significance for the uterus. Additionally, no studies discussed in this paper are able to model or assess the true functionality of the uterus: embryo implantation and the ability to carry a pregnancy to term. Myometrial function is not analogous to endometrial function, and without adequate modeling of the functionality of the endometrium, no definitive conclusions can be made regarding the benefits of EVMP. Future preclinical studies involving embryo implantation and fetal development are necessary to determine the significance of EVMP for uterine transplant.

## Conclusion

*Ex-vivo* machine perfusion is a versatile modality with the potential to preserve and optimize uterine grafts prior to transplantation. While many of the studies on uterine EVMP have been unrelated to preservation or transplantation, historical protocols can be used to inform future perfusions, in terms of surgical technique, perfusion machine design, perfusate composition, and graft monitoring. Further preclinical studies are needed to determine optimal perfusion protocols, to model endometrial function, and to definitively show a benefit to EVMP as compared to the current standard of SCS.
